# Enhanced configurational sampling methods reveal the importance of molecular stiffness for clustering of oxygenated organic molecules

**DOI:** 10.1039/d5cp01931a

**Published:** 2025-10-16

**Authors:** Jaakko Kähärä, Lauri Franzon, Stephen Ingram, Nanna Myllys, Theo Kurtén, Hanna Vehkamäki

**Affiliations:** a University of Helsinki Helsinki Finland hanna.vehkamaki@helsinki.fi

## Abstract

Oxygenated organic molecules (OOMs), formed in the atmosphere by oxidation of volatile organic compounds, have been speculated to take part in new particle formation (NPF). The key parameters for assessing the role of OOMs in NPF are the evaporation rates of small clusters, particularly dimers, derived from quantum chemical binding free energies. The main bottleneck for modelling OOM clusters is the conformational sampling of their high-dimensional potential energy surfaces. In this work, we update previous cluster conformational sampling protocols and apply them to OOM clusters. In addition to tuning cut-off energies and filtering approaches, we force hydrogen bond formation between molecules in the initial sampling, and use metadynamics simulations to search for additional minima. We compute dimer binding free energies for 104 dimers of accretion products formed in isoprene and toluene oxidation, 3 dimers of accretion products from alpha-pinene oxidation, and 36 dimers of polyethylene glycol molecules (PEGs). The binding free energies of the OOM homodimers are almost uncorrelated with the saturation vapour pressures predicted by existing group-contribution approaches. Also, the binding free energies are too high for substantial clustering in typical lower-tropospheric conditions. Using the PEG molecules, we demonstrate that both the weak binding, and the lack of correlation between binding free energies and saturation vapour pressures, are likely caused by intramolecular hydrogen bonding. This self-bonding is dictated by the molecular flexibility, which is ultimately a unimolecular property, and potentially a cost-effectively computable descriptor for assessing the clustering ability of OOMs.

## Abstract 2

Calculations on polyethylene glycol (PEG) clusters demonstrate the effect of self-bonding on cluster stability. Stiff molecules unable to self-bond (represented here by the hypothetical linear PEG monomers) form considerably stabler clusters.

## Graphical abstract

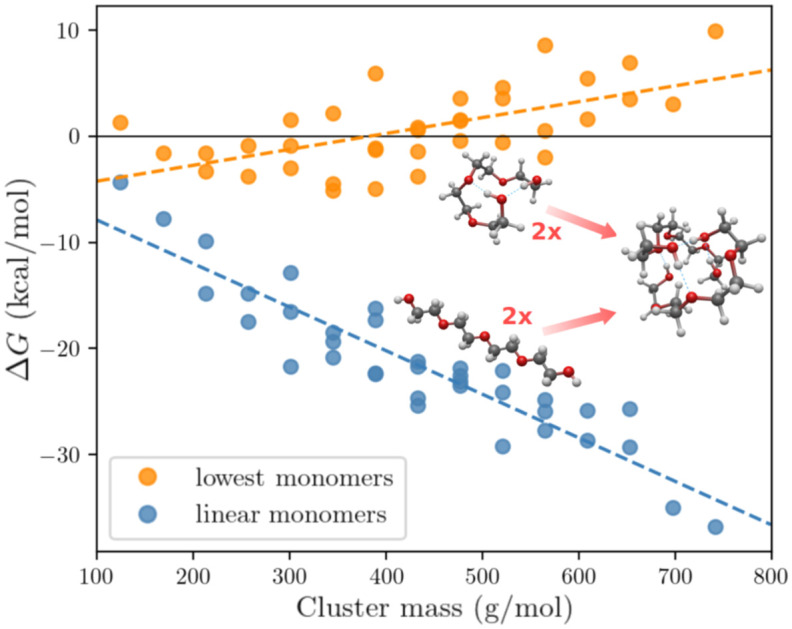

## 1 Introduction

Atmospheric aerosol formation in general, and secondary organic aerosol (SOA) formation in particular, has been the subject of intense research due to its effect on both air quality and global climate. SOA is composed mostly of oxygenated organic molecules (OOM), which form in the atmosphere by oxidation of volatile organic compounds (VOCs) emitted by both natural and anthropogenic sources. However, the very first steps in the formation of new aerosol particles (new-particle formation, NPF) are still poorly understood. A particularly intriguing open question is the relative contribution of OOMs compared to inorganic acids, bases and ions, first to the initial clustering, and then to the subsequent growth. Highly oxygenated organic molecules (HOM, a subclass of OOM, see *e.g.* Bianchi 2019 for precise definitions^1^) have been predicted to take part at least in the growth of newly formed atmospheric clusters to secondary atmospheric aerosol particles. Recently, isoprene-derived OOM were reported to form particles (“nucleate”) in the upper troposphere above the Amazon,^2^ possibly even without participation of inorganic acids or ions.

Accurate modeling of atmospheric NPF involving HOMs in realistic conditions has until recently been hampered by a lack of data on the precise molecular structures of the likeliest HOM candidates, as well as the large diversity of potential HOM compounds. These issues have been particularly severe for the compound classes most likely to initiate NPF: accretion products containing more carbon atoms than the original VOC precursors. A combination of experimental and modeling work has recently begun to alleviate both of these problems,^1,3,4^ and large datasets of structures of atmospherically realistic HOM accretion products can now be generated automatically.^5^ However, there are still some limitations in terms of the included chemistry, and another substantial restriction for modeling organic NPF still persists: the lack of reliable thermodynamic data.

Traditionally, atmospheric NPF has been modeled based on macroscopic thermodynamic properties, in particular saturation vapour pressures (*p*_sat_).^6–8^ However, this approach may be inappropriate in the context of HOMs for several reasons. First, experimental data on *p*_sat_ for complex polyfunctional atmospherically relevant organics, or indeed for almost any organic compounds with low enough *p*_sat_ to permit particle formation in atmospheric conditions, do not exist.^9^ Second, due to the conditions in which they form, and their high reactivity, HOMs are not stable in bulk quantities and cannot be synthesized using traditional laboratory methods. Third, while *p*_sat_ can be modeled by a variety of approaches, ranging from empirical parametrisations to sophisticated quantum chemistry – based models, all these approaches have large uncertainties when applied to realistic HOM candidates. Different modeling approaches often also disagree substantially with each other.^10^ Fourth, it is well-known from inorganic NPF studies that bulk properties, such as saturation vapour pressures, are relatively poor predictors of the actual parameters governing NPF rates: the evaporation rates of small clusters.^11^

Evaporation rates can be computed from quantum chemical cluster formation free energies using the detailed balance approach, and applying well established parametrisations for the collision rates. Combined with cluster population dynamics models, the collision and evaporation rates can then be used to predict NPF rates at given vapour concentrations or vapour formation rates and temperatures.^12^ While the collision rates of neutral small molecules with each other typically vary by less than a factor of 10, the evaporation rates easily span tens of orders of magnitude, meaning that the major uncertainties in modelling NPF stem from the evaporation rates.

Formation free energies for clusters of two or more HOM molecules (especially accretion products) are thus urgently needed, both to verify how well existing saturation vapour pressure modeling methods are capable of predicting both relative and absolute cluster stabilities, and to further narrow down the range of HOMs participating directly in NPF. The first step in computing formation free energies is conformational sampling, *i.e.* exploration of the possible three-dimensional structures of the involved molecules and clusters, typically with the objective of finding one or a few representative structures with as low (free) energies as possible (often called the “global minima”). Unfortunately, the large size and flexibility of aerosol-relevant OOM/HOM molecules, and the corresponding massive number of potential configurations, makes conformational sampling extremely time-consuming.

Methods and complications regarding configurational sampling have been discussed in detail in previous studies.^13,14^ The configurational sampling approach used in this work was implemented using the JKCS program^13^ which has been successful for finding global minima of clusters of small molecules and ions (H_2_SO_4_, NH_3_, *etc.*).^14^ However, the original approach in JKCS for generating initial guesses uses the ABC algorithm (ABCluster^15,16^) which we have found to be too inefficient when applied to large organic molecules. The primary issue is that, compared to small acid and base molecules, OOMs have far fewer possible orientations in which donors or acceptors may form hydrogen bonds relative to the total number of possible configurations. The flexibility of larger molecules also increases the size/dimensionality of the potential energy surface (PES) that needs to be explored. Furthermore, the semiempirical methods used for initial sampling often lead to changes in the covalent bonding patterns, *i.e.* chemical reactions. As the objective is to sample clusters of the original intact molecules, reacted structures must be identified and removed. Improved filters are thus needed to select configurations for studies with higher accuracy methods.

Our main goal in this paper is to improve the efficiency of sampling of OOM-clusters. This is achieved with the following additions to the JKCS sampling workflow: (1) during initial sampling, constraints are added to structure optimization to force the molecules to form a maximal number of H-bonds. (2) New filters are introduced to more efficiently select the most likely minimum energy configurations. (3) Metadynamics simulations (CREST^17^) are used to search for lower minima.

Experimental research suggests that OOMs with 15–20 carbon atoms can contribute to atmospheric cluster formation.^18^ However, generating sufficient amounts of accurate data even for dimer (two molecule) clusters of such compounds is computationally prohibitively expensive. Thus we have studied 50 C10–C14 sized OOM accretion products from isoprene and toluene oxidation, their homodimers (clusters of two identical molecules) and 54 OOM_A_–OOM_B_ heterodimers (mixed two molecule clusters). To understand the observed lack of correlation between the saturation vapour pressure and dimer formation free energy for the OOM clusters, we also studied a simpler test case of dimers formed of polyethylene glycol molecules (PEGs).

We note that for accurately modelling the effect of organic molecules *e.g.* on the growth of larger aerosol particles, we would need data not only on organic–organic interactions, but also on organic–inorganic interactions, especially the interactions with water molecules. These may introduce competitive binding effects, which further complicate the picture. However, in the context of gas-phase OOM molecules forming small clusters in atmospheric conditions, the role of water molecules is likely to be limited, as even the most hygroscopic organic species are predominantly found in their unhydrated forms^19^ due to the entropy penalty of clustering.

## 2 Methods

### 2.1 Molecule generation

To test our conformer sampling methods for larger molecules, we needed a set of example OOM molecular structures. As our main goal is to develop methods for modelling clustering of organic molecules, these example molecules should ideally be likely contributors to pure organic clustering. As such, the following criteria were chosen for molecule selection:

(1) The molecules must be organic accretion products from peroxy radical recombination (RO_2_ + RO_2_) reactions, as these are expected to be the least volatile gas phase organic molecules formed in the troposphere.^20^

(2) The molecules must be formed by the oxidation of common atmospheric VOC compounds.

(3) The molecules must have a sufficiently low saturation vapour pressure (*p*_sat_) at 298.15 K suggesting efficient clustering.

In addition, to ensure the computational feasibility of our calculations, two more criteria were chosen to restrict the size of the molecules:

(4) The precursor VOC forming the peroxy radicals (RO_2_) must have less than 10 carbon atoms, *i.e.* they may not be monoterpene-sized.

(5) The accretion products may have at most two functional groups containing oxidized N atoms. A similar criteria was used by Besel *et al.*^21^ in their molecule selection.

The GECKO-AP software^5^ was used to find a selection of molecules fulfilling the above criteria, specifically criterion 1. The accretion products generated by the code were both of the commonly known ROOR type and of the recently discovered ROR/R(O)OR type.^22^ Based on criteria 2 and 4, isoprene and toluene were chosen as precursor molecules when generating RO_2_ lists. For isoprene, up to 4th generation RO_2_ were generated, resulting in a total of 835 RO_2_ forming 33 940 RO_2_ + RO_2_ accretion products. For toluene, up to 4th generation RO_2_ were generated, resulting in a list of 4772 RO_2_ forming 661 191 accretion products. Saturation vapour pressures for the molecules were calculated using the Nannoolal^23^ and SIMPOL^24^ methods implemented in GECKO-A.^25^

Different *p*_sat_ cutoffs were chosen for the isoprene and toluene products due to vastly larger amount of the latter: for isoprene products a cutoff value of 10^−13^ atm was used, whereas a cutoff of 10^−15^ atm was used for the toluene products. With these cutoff values, the amount of accretion products fulfilling both the N atom and *p*_sat_ criteria was then 6308 for isoprene and 380 937 for toluene. Chemical heterogeneity was calculated for the remaining products, using the pairwise Tanimoto similarities^26^ of the topological fingerprints.^27^ The 20 most heterogeneous isoprene-derived and 30 most heterogeneous toluene derived molecules were then chosen for calculations in the next step. The SMILES strings and predicted *p*_sat_ values of the chosen molecules are listed in Table S2 of the SI. To access the full radical and product lists generated from GECKO-A see Data availability.

### 2.2 Sampling workflow overview

Our workflow for cluster sampling is implemented using the JKCS program^13^ which provides tools for data handling and easy interfacing with different quantum chemistry programs. The workflow efficiency has been improved compared to our previous studies. In particular, initial cluster generation has been optimized for oxygenated organic molecules, and cluster filtering has been refined to minimize the number of calculations needed during the cluster sampling. For a more detailed description of each computational step, see Section S1 of the SI.

Our central assumption is that most of the binding energy is determined by the number of hydrogen bonds and that the lowest lying minima are likely to be found by selecting the clusters with high number of H-bonds. While van der Waals interactions also contribute to the total energy, their strength is an order of magnitude weaker and we shall ignore them in the initial sampling.

Instead of the default ABCluster sampling, the initial set of sampled clusters are optimized using sets of constraints which maximize the number of H-bonds between molecules. Each donor is initially paired with only one acceptor, though additional H-bonds can form during xTB optimization. A subset of results with the highest number of hydrogen bonds are then selected for DFT optimization.

**Fig. 1:**
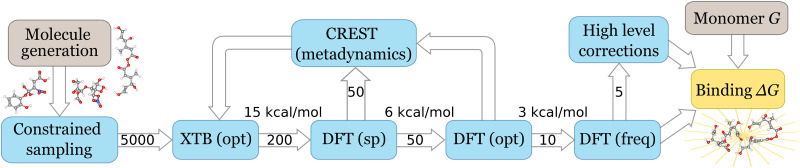
Configurational sampling workflow used in this paper. Filters (arrows) are applied between each computational step. The filtering thresholds *E*_lim_ are shown above the arrows, and the maximum number (*N*_lim_) configurations passed to the next step is shown inside each arrow. High level corrections may involve a single-point calculation, or re-optimization of the structure.

The workflow (illustrated in Fig. 1) for finding minimum energy structures is as follows:

(1) Constrained (forced H-bonding) sampling of up to 10 000 initial clusters (the total number depends on the number of unique H-bond combinations) using the GFN-FF forcefield.

(2) Relaxed (*i.e.* without constraints) re-optimization at semi-empirical GFN1-xTB level.

(3) Filter out reacted clusters (including proton transfers) and duplicates.

(4) Single-point (SP) electronic energy calculations using a cost-effective density functional theory method (here, ωB97X-D3/def2-SVP), and select the lowest energy clusters.

(5) Look for lower energy structures using the CREST software (at GFN1-xTB level). Repeat single-point ωB97X-D3/def2-SVP calculation.

(6) Loose geometry optimization using ωB97X-D3/def2-SVP (same level as in step 4).

(7) If needed, repeat steps 5–6 to find even lower energy structures.

(8) Low level (tight) geometry optimization, frequency and free energy calculation using ωB97X-D3/def2-SVP.

(9) Optionally, calculate a SP correction or re-optimize using a higher level of theory (here, ωB97X-D3/def2-TZVP) to obtain final Gibbs free energy. (For OOMs, a final SP correction was calculated with the ma-def2-TZVP basis set.)

The initial constrained sampling was performed using the ABCluster software,^15,16^ whereas the xTB optimizations are done using the dedicated XTB software.^28^ Instead of the default ABCluster sampling, the initial set of sampled clusters are optimized using sets of constraints where each donor is initially paired with only one acceptor. This typically maximizes the number of H-bonds between molecules (though additional H-bonds can form during xTB optimization). A subset of results with the highest number of hydrogen bonds are then selected for DFT optimization.

All DFT calculations were performed using ORCA version 6.0.^29–33^ We picked the ωB97X functional, with the D3 dispersion correction, as this functional has shown excellent performance in benchmarking comparisons, and has been extensively used in previous atmospheric cluster studies.^34,35^ At each step of the workflow, we determine which configurations have so high energy that they are very unlikely to optimize to the global minimum. Thus, we only select cluster configurations whose energies *E* are within the range*E* ≤ *E*_min_ + *E*_lim_,where *E*_min_ is the energy of the current lowest lying configuration, and *E*_lim_ is the cut-off limit. Additionally, for systems with particularly large numbers of cluster configurations a maximum number of configurations (*N*_lim_) was taken to the next step of the workflow. Table 1 lists the filtering thresholds *E*_lim_ and *N*_lim_, and approximate number of cluster configurations produced and computation times at each step of the workflow. Based on the initial results, we were able to determine energy correlations between workflow stages (see Fig. S1) and adjust the thresholds to minimize the number of computations. In the case of the SP calculation step, *E*_lim_ was decreased from 8 to 6 kcal mol^−1^ after the initial testing.

**Table 1:** Approximate cluster counts and wall clock times at each step of the sampling workflow used to produce dimers of PEGs and OOMs (C10–C14 sized molecules). Filtering thresholds *E*_lim_ are applied after the calculations. If more than *N*_lim_ are within cutoff after filtering, *N*_lim_ lowest energy clusters are selected. Low DFT corresponds to ωB97X-D3/def2-SVP and high DFT to ωB97X-D3/def2-TZVP level of theory. PEGs were optimized up to low DFT, and OOMs up to high DFT

Method	*N* clusters	Time/cluster	*E*_lim_ (kcal mol^−1^)	*N*_lim_
ABCluster (GFN-FF)	10 000	1 s (1 cpu)	25	5000
GFN1-xTB	5000	15 s (1 cpu)	15	200
CREST	20–50	4 min (8 cpu)		
Low DFT (single-point E)	100–200	3 min (40 cpu)	6	50
Low DFT (optimization)	10–50	2 h (40 cpu)	3	10
Low DFT (frequencies)	3–10	30 min (40 cpu)	3	5
High DFT (single-point E)	3–5	8 min (40 cpu)	3	3
High DFT (opt + freq)	1–3	4 h (40 cpu)		

#### 2.2.1 Cluster configuration filtering

To reduce computation time spent using more expensive methods, we want to filter out duplicates, reacted clusters, or otherwise unwanted structures such as those containing changes in *cis*–*trans* isomerism. A detailed overview of how duplicate cluster topologies are detected and filtered is provided in Section S1.4.

With respect to reactions, genuine proton transfers commonly occur when acids and bases (for example H_2_SO_4_ and NH_3_) form clusters. In JKCS these are typically handled by running separate configurational samplings with different protonation states.^14^ However, our OOM species lack basic (proton accepting) functional groups, so proton transfers are not anticipated. Nevertheless, both proton transfers and other unwanted reactions sometimes occur during the initial semi-empirical optimization, or at the start of DFT optimization for high-energy configurations. Reactions usually result in a significant jump in energy, but there are many exceptions, and detecting a reacted cluster is not always straightforward. In this work we subsequently filtered out all detected reacted states.

We use the atomic simulation environment (ASE)^36^ to systematically probe interatomic distances. We compare the bond lengths inside the generated clusters to those in the original molecules and consider a cluster configuration as reacted if any bond length has changed more than a pre-defined tolerance value. Because some covalent bonds, such as O–O, can stretch quite easily and may also vary between different levels of theory, the relative tolerance has to be fairly high (in this work, we set it at 20%). Additionally, if two previously non-bonded atoms are found too close to be classified as H-bonds (H⋯O distance <1.4 Å) or other non-covalent interactions, we can assume that a reaction has occurred.

Finally, we also filter out configurations that are unlikely to optimize to the global minimum. This is done by constraining the electronic energies and hydrogen bond counts. If after filtering the number of clusters remains higher than the available computational resources, we select a set number of lowest energy clusters.

#### 2.2.2 Monomer conformer sampling

Our cluster sampling workflow can also be used to efficiently sample the configuration space of gas phase monomers. Using constraints provides a very fast method for generating initial set conformers and reduces the time needed to spend on metadynamics simulations. Filtering parameters were generally the same as for clusters, though the energy thresholds *E*_lim_ were decreased. Due to the potential energy surfaces of single molecules being inherently less complex, we believe the lowest energy structures found for monomers are more likely to correspond to the actual global minimum than is the case for dimers.

We used rdkit^37^ to generate the initial coordinates for each monomer isomer from the SMILES strings. Because stereoisomerism is currently not defined for the molecules generated by Gecko-AP, we performed conformer sampling for all *cis*–*trans* isomers of C=C – containing monomers. Cluster sampling and binding energy calculations were then performed using only the lowest energy isomers. The existence of double C=C bonds can severely restrict the flexibility of a molecule, and depending on the *cis*–*trans* isomerism it can result in significantly different lowest-energy conformers. We do not expect *cis*–*trans* isomerism to change spontaneously in atmospheric conditions due to the high barriers involved. Therefore, an additional filter was implemented which determines the isomers inside clusters (using rdkit), and removes clusters where the isomerisms does not match the lowest-energy isomer (*i.e.* where the conformation sampling of the clusters has erroneously led to rotations around C=C bonds). We assume that the choice of chirality does not affect the energy.

## 3 Results and discussion

Our initial dataset for the workflow development consisted of 50 OOM – homodimers, built from the C10–C14 sized monomers (see Table S2) described in Section 2.1. We then further picked 12 OOM corresponding to some of the strongest and weakest-bound homodimers, and attempting to maximise structural diversity, and constructed 54 heterodimers from them. An additional 36 dimers were constructed from the 8 smallest polyethylene glycols (PEGs) to understand the observed binding trends. Finally, the developed conformational sampling methods were used to construct three clusters from two C20 – sized monoterpene-derived OOMs, which will be discussed in Section 3.4.

**Fig. 2:**
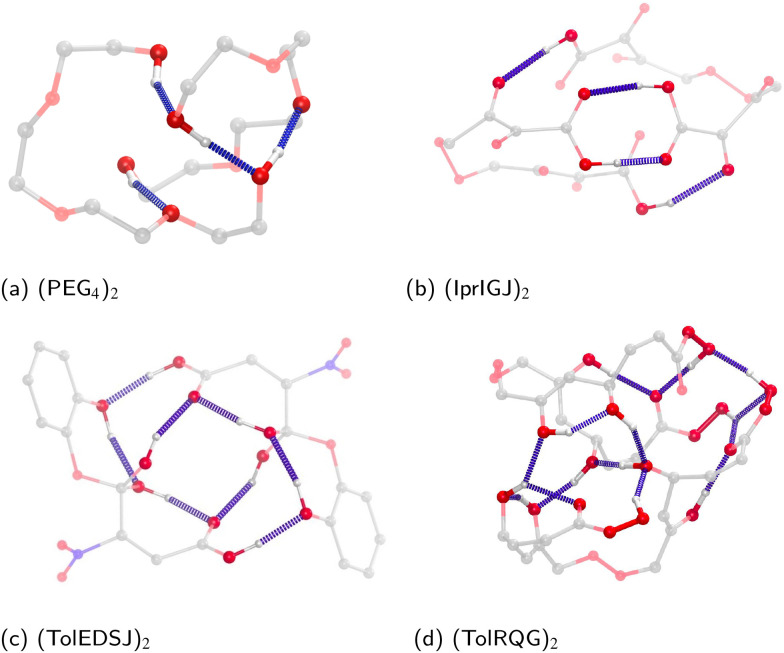
Some examples of lowest free energy cluster configurations, with hydrogen bonds highlighted in blue. Note that hydrogens bonded to carbons have been omitted.

Examples of lowest energy OOM-dimers shown in Fig. 2, with the detected hydrogen bonds highlighted (non hydroxyl protons are excluded for clarity). We find that the global minima tend to adopt conformations that are spherical or “globular”, rather than the two monomers bonding one another in a linear arrangement. Cyclic (or interconnected) arrangements of H-bonds are often favoured, especially those that would promote facile interchange of donor and acceptor groups; for example, two carboxylic acid groups forming a six-membered ring (panel b), or a chain of eight H-bonds arranged in a spiral like configuration (panel c). More generally, there seems to be a trade-off between intra- and intermolecular H-bonds forming, with both usually being present in low energy dimer structures.

### 3.1 OOM homodimer binding free energies

**Fig. 3:**
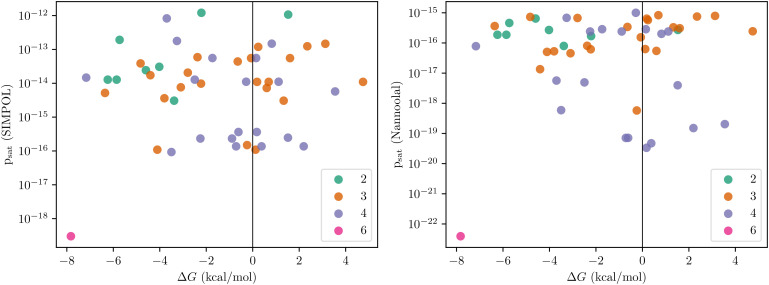
Binding free energies of 50 homodimers (OOM_2_) at 298.15 K and 1 atm reference pressure v. SIMPOL and Nannoolal saturation vapour pressures (in atm) of the OOMs. Colors indicate the number hydrogen donors in the corresponding OOM.

Traditionally, aerosol modelers assume that saturation vapour pressure (*p*_sat_) is a good measure of clustering ability, at least for organic molecules. With this in mind, we were surprised to discover that the predicted binding free energies Δ*G* of the 50 homodimers were almost uncorrelated both with the Nannoolal^23^ and SIMPOL^24^ predicted saturation vapour pressures. The saturation vapour pressure predictions also disagree with each other, in some cases by more than three orders of magnitude – in line with previous studies, for example.^10^ The relation of Δ*G* values of homodimers to *p*_sat_ values and the number of H-bond donors has been illustrated in Fig. 3. Indeed, aside from one data point, the most strongly binding dimers show higher saturation vapour pressures than the weakest. There is also no obvious correlation between the number of detected hydrogen bonds within the cluster (colour scale) and either binding energy or vapour pressure. Furthermore, despite almost all of the studied OOM being extremely low volatility organic compounds (ELVOC) based on their saturation vapour pressures, the Δ*G* values are far too high (insufficiently negative) to permit clustering at atmospherically realistic (sub-ppt) mixing ratios, at least at the temperature of 298 K.

For a generic homodimer formation reaction M (monomer) + M (monomer) ⇔ D (dimer), the equilibrium mixing ratio of dimers (*p*_D_/*p*_ref_) can be expressed as exp(−Δ*G*/(*RT*)) × (*p*_M_/*p*_ref_)^2^, where *p*_D_ and *p*_M_ are the partial pressures of the dimers and monomers, respectively, and *p*_ref_ is the reference pressure at which Δ*G* is calculated (here, 1 atm). Note that the nominal pressure dependence of Δ*G* arises from the translational component of the entropy, (see, *e.g.* Ochterski^38^ for details). Given monomer mixing ratios (*p*_M_/*p*_ref_) on the order of 10^−12^, even the lowest predicted Δ*G* value of −8 kcal mol^−1^ correspond to dimer mixing ratios on the order of 10^−19^ (or below ten clusters per cm^3^, *i.e.* around one in a million monomers is found in a cluster).

Shen *et al.*^2^ suggest that isoprene oxidation products are able to nucleate on their own (without inorganic acids or ions) in the considerably colder conditions found in the upper troposphere. As 20 of our OOMs originate from isoprene oxidation, a more direct comparison with their data is warranted. Tables S3–S5 in the SI show all the OOM-dimer binding free energies in this study recomputed at a temperature of 223.15 K (−50 °C), corresponding to the experiments in the Shen *et al.*^2^ study. At this temperature, the most strongly bound OOM homodimers have Δ*G* values on the order of −12 kcal mol^−1^, and a substantial fraction are around −10 kcal mol^−1^. Using monomer mixing ratios on the order of 10^−12^ (corresponding quite well to the 10^6^–10^7^ molecules per cm^3^ range in the Shen *et al.* study^2^), we now obtain dimer mixing ratios close to 10^−13^, or about one-tenth of the monomers. While evaporation of dimers is still more likely than collision with monomers, the trimer production rate ([D] × [M] × collision rate) is now of the same order as the nucleation rates measured by Shen *et al.* study.^2^ Thus, if the evaporation rates of trimers and larger clusters were negligible compared to their growth rates, the OOM data presented here would not rule out pure organic nucleation of isoprene-derived accretion products at −50 °C. However, the assumption of negligible evaporation of trimers may be unrealistic, and should be tested in future studies.

### 3.2 The PEG dimer test case

These results indicate that *p*_sat_ may not be as effective a measure of OOM clustering ability as has been generally assumed, as evaporation from a bulk liquid surface is not comparable to evaporation from a small cluster of a few molecules surrounded by gas phase.

To determine whether the lack of correlation between Δ*G* and *p*_sat_ for the OOMs is a modelling artefact or a real result, we then moved to simulating dimers of polyethylene glycols (PEG_*n*_ = H–(O–CH_2_–CH_2_)_*n*_–OH, *n* = 1, 2, …, 8). These systems have three advantageous features. First, their saturation vapour pressures have been experimentally measured. Second, previous studies have demonstrated that quantum chemistry – based methods (in particular, COSMO-RS), are capable of at least qualitatively predicting the behaviour of their saturation vapour pressures as a function of size of the molecule.^39^ Third, since each PEG molecule only has two H-bond donors (the OH-groups at the ends), we can efficiently sample all possible H-bond combinations, and be reasonably confident of finding the global minimum structure. Our computed Δ*G* values for the PEG clusters will thus not suffer from errors related to possibly incomplete conformational sampling.

**Fig. 4:**
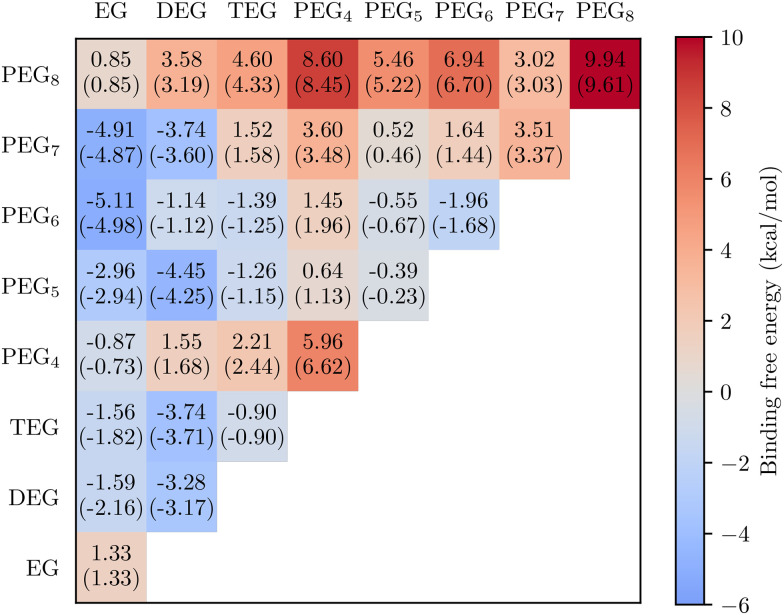
Cluster binding free energies for (PEG_*n*_)(PEG_*m*_)-dimers up to *n*, *m* = 8. The lowest formation free energy minima are accompanied in brackets with the values Boltzmann averaged over a set of low lying free energy configurations. Temperature is 298.15 K and reference pressure 1 atm.

**Fig. 5:**
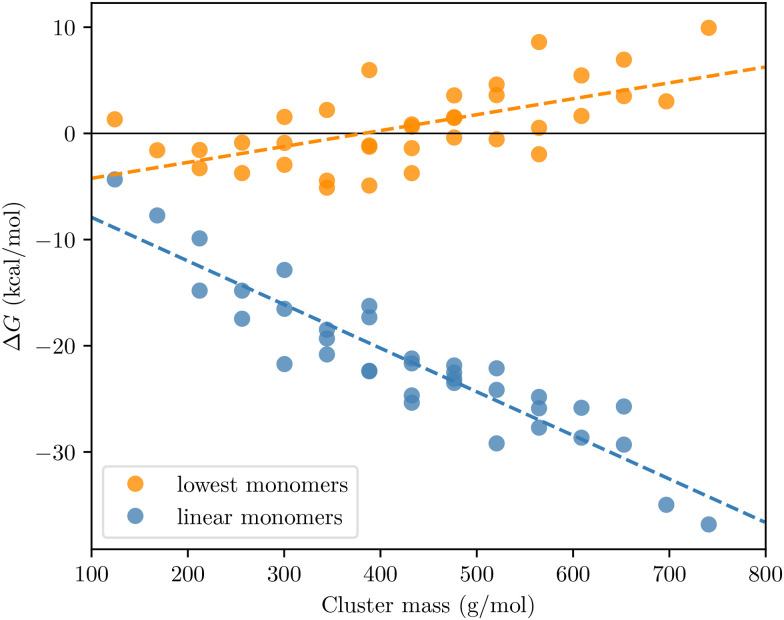
PEG dimer binding free energies v. Cluster size calculated with the lowest free energy monomers, and artificial linear monomers. Cluster configurations are same in both cases, only monomer configurations are different. Temperature is 298.15 K and reference pressure 1 atm.

The PEG-dimer binding free energies, calculated at 298.15 K, and reference pressure 1 atm, are shown in Fig. 4. Based on both measured and quantum chemistry – derived saturation vapour pressure values,^39,40^ we would expect Δ*G* to decrease when PEG sizes increase. Instead, we observe the completely opposite pattern: the predicted Δ*G*'s in fact increase slightly with increasing molecular mass (orange points in Fig. 5), counter to the trend of vapour pressures. Test calculations using multiple conformers (and Boltzmann-averaging) to compute the free energies did not change this pattern (see values in brackets in Fig. 4). We can thus conclude that the unexpected trend in OOM dimer Δ*G*'s is not due to incomplete conformational sampling.

If we optimize the geometries of the PEG monomers starting from an artificial linear structure, and use these linear monomers instead of the lowest free energy conformers as the reference point for calculating the binding free energy, the expected decreasing trend is recovered (blue points in Fig. 5). In fact, the trend in equilibrium constants for dimer formation (proportional to exp(−Δ*G*/(*RT*))) is now much stronger than the trend in *p*_sat_: from PEG_1_ to PEG_8_, the former increases by over 20 orders of magnitude, while the corresponding decrease in *p*_sat_ is only about 8 orders.^40^

The dramatic difference between the two set of results in Fig. 5 is explained by the lowest free energy PEG monomers folding inwards onto themselves, and forming intramolecular hydrogen bonds. The absolute free energy of the lowest free energy monomer state often decreases faster than the dimer free energies as the molecules become larger. We note that PEG_4_ and PEG_8_ have unexpectedly strong internal H-bonds, and consequently the free energies of the dimers containing these PEGs deviate somewhat from the trends of the rest of the grid in Fig. 4. Due to the high computational cost at the level of theory used here, we did not continue the series up to PEG_12_, so it is unclear whether this trend continues.

Qualitatively, the volatility-increasing effect of intramolecular H-bonds is well-known, and can be experimentally observed as modest differences in bulk properties such as saturation vapour pressures between structural isomers with different H-bonding abilities. A classic example pair are 1,2-benzenediol (catechol), which can form intramolecular H-bonds, and 1,4-benzenediol (hydroquinone), which cannot.^41^ However, our results demonstrate that the effect is vastly stronger for dimer formation free energies than for saturation vapour pressures. Intramolecular H-bonding in the monomers is the underlying reason for why the Δ*G* values for the OOM clusters in Fig. 3 are so high (*i.e.* less negative than we would expect based on the saturation vapour pressures).

We emphasize that the linear monomers used here should be considered a diagnostic tool, or a hypothetical reference case. Real PEG or OOM monomers in the gas phase do form intramolecular H-bonds, and the orange points in Fig. 5, as well as the data points in Fig. 3, are the actual dimer binding free energies. However, in bulk solutions, the bonding environment will differ from that in the gas phase, and the effect of intramolecular H-bonds will be smaller. The linear monomers provide a useful reference for how the system would behave if the effect of intramolecular H-bonds were zero. As demonstrated by the quantitative mismatch between the size dependences of the equilibrium constants for the formation of dimers from “linear” monomers and the *p*_sat_ values, intramolecular H-bonding is not negligible even for bulk liquid systems: the actual liquid-phase behaviour is somewhere in between the two lines in Fig. 5.

### 3.3 OOM heterodimers

The previous sections demonstrate that due to intramolecular H-bonding, simple descriptors like saturation vapour pressures or the number of H-donors are not sufficient to predict the binding free energies of OOM clusters. The ability to form intramolecular H-bonds depends on the structure and flexibility of the molecule. For example, the existence of rings and double (C=C) bonds in the carbon chains of some OOMs, especially aromatic rings in some of the toluene-derived OOMs, is likely to impede the formation of intramolecular H-bonds. This can be observed from the fact that the lowest free energy conformers of many C=C – containing OOMs have unbonded (“hanging” or “dangling”) H-bond donors. Indeed, the most strongly binding molecules in our dataset all contain double bonds (and/or aromatic rings). However, this should not considered a general rule, as not all molecules with double bonds bind strongly: depending on which isomer we are sampling, double bonds may either hinder or promote internal hydrogen bonding.

The number of double bonds (not counting aromatic rings) is well-known to correlate with the ability of an organic molecule to form SOA. For example, limonene (with two double bonds) generally has higher SOA yields than most other monoterpenes (which typically have only one double bond). This has been explained in terms of the chemical reactivity: double bonds tend to increase both the rate of the initial oxidant attack, as well as the rate of subsequent autoxidation steps.^42^ While this undoubtedly remains the main explanation for the correlation, the present study suggests another potential contributing factor: oxidation products retaining double bonds may cluster more efficiently than otherwise similar molecules lacking such bonds. We note that this opens up interesting research questions concerning the subsequent chemistry. Molecules with double bonds are likely to be efficiently oxidized also within clusters, and the clustering may affect the rates and mechanisms of key reaction steps, as well as the competition between different reaction channels. In particular, the presence of clustering partners may increase the likelihood of bimolecular *versus* unimolecular reactions of reactive oxidation intermediates such as acyl peroxy radicals or Criegee intermediates, potentially leading to further accretion reactions.^1,43,44^ However, we note that most of the “double bonds” in the OOMs studied here belong to aromatic rings, which are not easily oxidized.

**Fig. 6:**
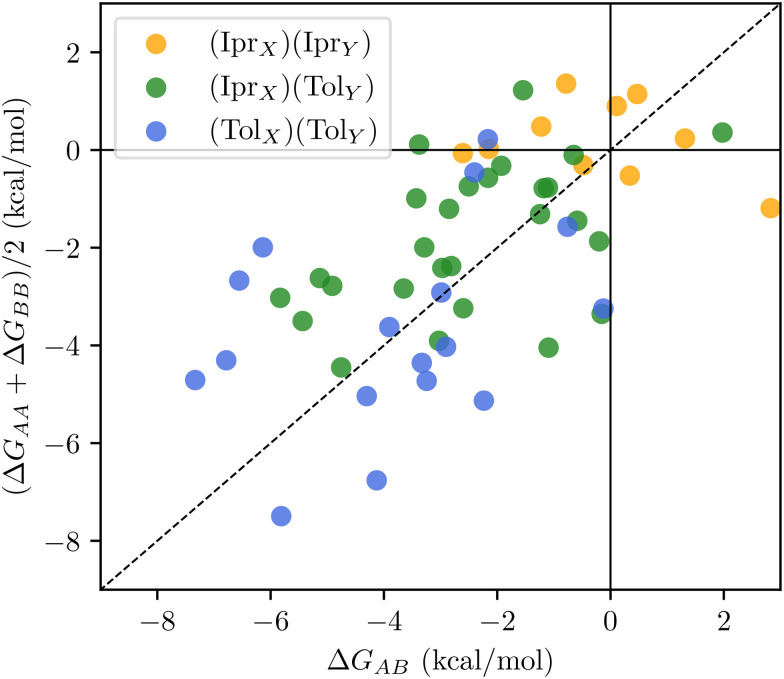
Binding free energies of heterogeneous (OOM_A_)_1_ (OOM_B_)_1_ clusters for selected isoprene (Ipr) and toluene (Tol) products, compared to the averages of homogeneous (OOM_A_)_2_ and (OOM_B_)_2_ cluster energies. Temperature is 298.15 K and reference pressure 1 atm.

While the strong dependence of the dimer binding free energies on monomer flexibilities prevents the use of descriptors based on bulk properties, it also paves the way for new approaches. In particular, the monomer flexibility is inherently a unimolecular property – its relative effect on the stabilities of different clusters containing the same monomer should be roughly similar. In other words, the effect should be transferable from one cluster environment to another. We tested this hypothesis on our set of 54 heterodimers by comparing their binding free energies (Δ*G*_AB_) to the average of the two corresponding homodimers (Δ*G*_AA_ and Δ*G*_BB_). As shown in Fig. 6, the correlation is fairly good: the prediction errors of the equation
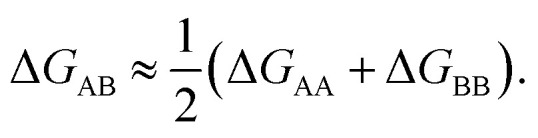
are MAE = 1.68 kcal mol^−1^, and RMSE = 1.99 kcal mol^−1^.

This correlation is useful in multiple ways. First, plots such as Fig. 6 serve as sanity checks for our results: if a data point diverges an unreasonable amount from the expected correlation, the sampling of one or more of the three involved cluster types (AA, BB and AB) has likely failed at finding a sufficiently low-lying minima. Second, unimolecular flexibility parameters or descriptors (for example, the difference in free energies between hypothetical non-H bonded and lowest free energy conformers) are much easier and less costly to compute than binding free energies of dimers or especially larger clusters. Currently, the application of, for example, machine learning methods to OOM cluster stabilities is prevented by the expense of the data generation. Even with the novel methods developed in this study, it remains computationally unfeasible to generate more than some hundreds of OOM clusters. In contrast, unimolecular properties can be calculated for tens of thousands of species, as demonstrated by Besel *et al.*^21^ We anticipate that a combination of simple functional group descriptors with “flexibility corrections” will provide a useful way of predicting binding free energies of OOM clusters in the future.

The fundamental role of molecular flexibility in determining dimer binding free energies has been studied at a more general level by Forrey, Douglas and Gilson.^45^ They performed molecular dynamics simulations on chains of Lennard-Jones beads with varying chain rigidity. Similar to our results, they find that increasing flexibility often leads to decreased dimer binding, both in the limit of extremely rigid molecules, and (due to self-interactions analogous to the internal H-bonds studied here) for extremely flexible long chains.

### 3.4 Dimers of monoterpene-derived accretion products

To test whether the phenomena we have described also apply to larger systems, we created new homo- and heterodimers containing two different monoterpene-derived accretion products, here labeled DA and DB. Both are peroxide (ROOR) – type accretion products, formed through RO_2_ + RO_2_ recombination reactions, with the RO_2_ produced from the oxidation of alpha-pinene.

DA (Fig. S4a) corresponds to the recombination product of one of the three 7-oxygen RO_2_ shown by Berndt *et al.*^46^ (we chose the species with the fastest computed route for the three-oxygen RO_2_ precursor, labeled “4” in their paper). This represents our current best guess of what highly oxidized accretion products from OH-initated alpha-pinene oxidation would look like. DB (Fig. S4b) corresponds to the product obtained from recombination of the two 8-oxygen RO_2_ from Iyer *et al.*,^47^ and represents our current best guess of what highly oxidized accretion products from O_3_-initiated alpha-pinene oxidation would look like.

**Fig. 7:**
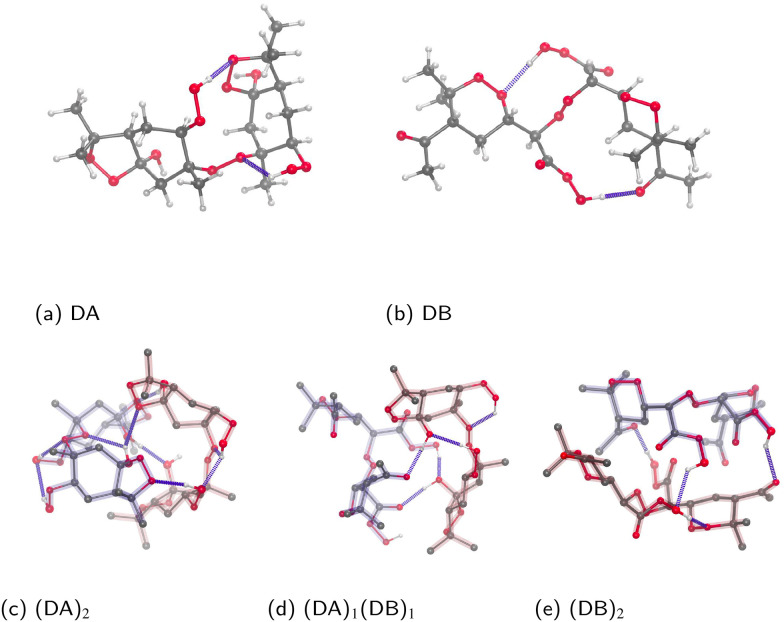
Monoterpene accretion products, labelled here as DA and DB, and their lowest lying dimer clusters of monoterpene products. In the clusters, molecules have been highlighted separately, and hydrogens bonded to carbons have been omitted for clarity.

Minimum energy structures and binding free energies were found for the two homodimers and one heterodimer, as in the previous subsections. The results are shown in Table 2, and illustrations of the lowest energy dimers are presented in Fig. 7. We note that, due to their formation pathways, these molecules contain hydroperoxide rather than hydroxyl groups. Based on experimental observations, we expect this to lead to, on average, stronger hydrogen bonding.^48^

**Table 2:** High level binding free energies (in kcal mol^−1^) of C20-sized molecule dimers at high and low temperatures (reference pressure 1 atm). For full results see Table S6

Cluster	*N* atoms	298.15 K	223.15 K
(DA)_2_	132	−5.86	−10.72
(DB)_2_	128	−8.86	−13.06
(DA)_1_ (DB)_1_	130	−5.15	−9.55

The binding free energies are comparable to the most strongly bound C10–C14 sized homo- and heterodimers. This is somewhat surprising, as the larger molecules could be expected to bind much more strongly. Furthermore, while DB only has two and DA four H-bond donors, they both have ring structures that hinder intramolecular binding, and could be expected to lead to greater cluster stability. It may be that the greater flexibility of the OOH groups allow for even more intramolecular H-bonding, decreasing the (relative) cluster stability. However, lacking a precise definition of molecular flexibility, this is difficult for us to quantify at present. We caution that the relatively large difference between the heterodimer and one of the two homodimers may indicate that the developed sampling workflow is still insufficient for these large molecules and their clusters. Thus, we cannot completely rule out the possibility that we have simply missed some substantially more strongly bound cluster structures.

Even with this caveat, the results in Table 2 do seem to indicate that pure neutral organic particle formation is rather unlikely in atmospheric, or at least lower-tropospheric conditions: the detailed-balance evaporation rates corresponding to the binding free energies are on the order of 10^5^…10^7^ s^−1^. This in turn implies that the clusters will evaporate very much faster than they collide with additional HOM molecules, which inevitably have mixing ratios in the sub-ppt range (corresponding to pseudo-unimolecular collision rates of around 0.01 s^−1^ or less). We note that this is in line with a large number of studies on simpler model compounds,^34^ which all predict negligible pure neutral organic clustering at atmospherically realistic temperature-concentration combinations.

## 4 Conclusions

We have shown that conformational sampling protocols developed either for single molecules, or for clusters of relatively simple monomers, can be extended to clusters of complex and flexible organic molecules, in particular oxidized organics relevant to atmospheric aerosol formation. However, this requires a combination of new computational approaches and researcher insight. Specifically, we suggest using both metadynamics to enhance the exploration of local minima, and H-bonding topologies to constrain the number of initial structures.

We have applied our new conformational sampling protocol to generate 50 homo- and 54 heterodimers of C10–C14 sized accretion products from isoprene and toluene oxidation, 36 dimers of polyethylene glycol (PEG) model compounds, and 3 dimers of C20-sized monoterpene-derived accretion products. Surprisingly, the binding free energies of the atmospherically relevant products were correlated neither with their predicted saturation vapour pressures, nor with the number of H-bonding functional groups. With the help of the PEG dimer data, we show that this can be explained and understood in terms of the intramolecular H-bonding in the monomers, which reduces the relative stability of the clusters.

This observation leads to two conclusions. First, the molecular (in)flexibility is likely to be a strong factor in determining the clustering efficiency of organic oxidation products, much more so than predicted bulk saturation vapour pressures. Compounds containing, for example, double bonds or rings are likely to be more efficient at clustering than otherwise similar molecules lacking these features. As molecules containing double bonds will likely be further oxidized also within the clusters, this has the potential to open up a new branch of atmospheric oxidation chemistry. Second, it should be possible to develop novel unimolecular descriptors for predicting this flexibility, and its effect on clustering, directly from the molecular structure (*i.e.* the stick figure or SMILES string). This would represent a cost-effective way of predicting the stability of complex organic clusters without the need to directly model the resultant cluster at a high level of theory. We believe these descriptors could be used to augment, for example, the cluster-of-functional groups approach for predicting cluster formation free energies.^49^

Finally, while the organic cluster dataset presented here is still relatively limited, it represents yet another negative result in terms of finding a highly oxygenated organic molecule capable of pure neutral NPF under tropospheric conditions. It is possible that organic NPF proceeds *via* some other mechanism than simple H-bonded cluster formation, such as gas- or cluster-phase oligomerization. However, we note that the molecules studied here have already undergone accretion reactions, and are thus considerably larger than the original precursors. It is quite remarkable that even clusters of two C20 – sized molecules seem to be unstable in tropospheric conditions. Our results suggest that, if organic only nucleation by clustering does occur, it will involve HOMs that are relatively inflexible and non-self bonding. We recommend focusing efforts on new methods to discriminate between flexible and inflexible HOMs.

## Author contributions

JK: cluster sampling, writing filtering code, data analysis, visualization, and drafting the paper. LF: selecting OOMs for calculations. SI: supervision of research and drafting the paper. NM: selecting PEGs as model system. TK, and HV: research question design and iteration. All authors took part in writing parts of the manuscript, revising and commenting the whole manuscript.

## Conflicts of interest

There are no conflicts to declare.

## Supplementary Material

CP-027-D5CP01931A-s001

## Data Availability

Molecular definitions, and binding free energies of clusters generated in this work have been provided in the supplementary information (SI). See DOI: https://doi.org/10.1039/d5cp01931a. The DFT optimized monomer and cluster data, including ORCA output files, and the GECKO-A radical and product lists are available at a Fairdata repository: https://doi.org/10.23729/fd-49d97a04-8022-3f20-91be-1465902305ba.
