# Supplementary material for: Enhanced configurational sampling methods reveal the importance of molecular stiffness for clustering of oxygenated organic molecules
Source: Phys Chem Chem Phys. 2025 Oct 16;27(43):23410–20. doi: 10.1039/d5cp01931a (PMC12542396; doi:10.1039/d5cp01931a)
Supplement: CP-027-D5CP01931A-s001 [file CP-027-D5CP01931A-s001.pdf]

Supplementary Information (SI) for Physical Chemistry Chemical Physics.
This journal is © the Owner Societies 2025

Supplementary material

Jaakko Kähärä, Lauri Franzon, Stephen Ingram, Nanna Myllys,
Theo Kurtén, Hanna Vehkamäki

S1 Stages of the sampling workflow

S1.1 Initial configuration generation

Our central assumption informing the configurational sampling of OOM clusters is that the global minimum configuration is located relatively close to the maximally H-bonded configuration on the potential energy surface. Informed by this assumption, we start our search for low-energy configurations by only sampling configurations with at least as many H-bonds as hydrogen donors. However, depending on the structure and flexibility of the molecules, they may not be able to efficiently form intermolecular hydrogen bonds, and we must settle for searching for the highest plausible number of H-bonds.

To illustrate the number of possible cluster configurations, we can assume two molecules both of which contain N_H hydrogen donors, and $N_O \geq N_H$ oxygen acceptors (and thus, N_H oxygens belong to an OH group). We exclude combinations where hydrogen bonds are formed between two OH groups in a four-membered ring structure i.e. where both oxygens act simultaneously as donor and acceptor. If we force each donor to bond with one acceptor of the opposing molecule, the total number of possible H-bond combinations is

$$\left(\frac{N_O!}{(N_O - N_H)!}\right)^2 .$$

The number of combinations becomes even higher if the donors are able to form a bond with more than one acceptor, or vice versa. However, if the molecules are identical or contain symmetries, some of the combinations are non-unique.

The total number of H-bond combinations is often too high for practical purposes. If the number of combinations is above 5000, we select a random subset. We can also reduce possible combinations by assuming that the strongest binding energy is obtained by pairing donors and acceptors with the highest partial charges. In our case, H-donors were only paired with oxygen atoms in other OH/OOH-groups or doubly bonded oxygens (C=O, or N=O in $-NO_2$ and $-ONO_2$ groups). N$\cdots$O dipole-dipole interactions were also counted for monomers containing nitrate ester $-ONO_2$ groups. The nitrogen atom in these has a high partial charge (>0.7), and may form intermolecular bonds with oxygen atoms in other groups with a N$\cdots$O bond.

Bond counting alone is not sufficient to predict which cluster configurations have the lowest energies, because different bond combinations may have an extremely high variation in energy. Nevertheless, the sets of maximally H-bonded cluster configurations generated by this approach provides an excellent starting point for further configurational sampling.

S1.2 Constrained Sampling

The JKCS workflow begins with ABCluster [1;2] which using a genetic algorithm searches for minimum cluster configurations and optimizes the configurations with the XTB program. (The ABC algorithm places the monomers at random initial locations in a box, and iteratively searches for the global minimum.) This is a viable task for clusters of small molecules like water and sulphuric acid, but when applied to highly flexible molecules with millions of possible conformers, the genetic algorithm becomes very inefficient. In practice, the monomers are generally not oriented favourably enough to form hydrogen bonds and tend to fly apart during optimization. We found that less than 10% of sampled initial configurations optimize into a cluster with at least one H-bond between molecules. To

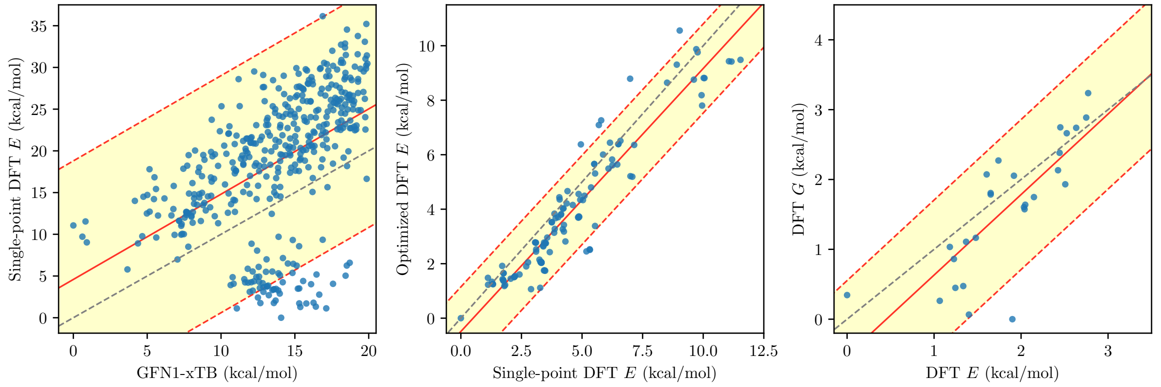

Figure S1: Example correlations at different cluster sampling steps for the $(\mathrm{TolA})_2$ cluster (for SMILES see Table S2). The highlighted areas correspond to where 95% of values would fall, assuming a Gaussian distribution. (Axes have been shifted so that the lowest values are at 0. Free energies are calculated at 298.15 K and 1 atm)

address this issue, we modified ABCluster to force the monomers to form hydrogen bonds regardless of their initial positions.

Instead of the regular (relaxed) geometry optimization, we provide the XTB program (inside ABCluster) a list of as constraints which pairs each donor with one acceptor. This maximizes the number of H-bonds, equalling the total number of donors in the cluster. The constraints add an artificial harmonic force field between specified atom pairs to force their relative positions at a fixed distance (1.85 Å for H-bonds and 2.9 Å for possible N$\cdots$O interactions, based on IUPAC recommendations[3]). To prevent unwanted reactions, we used a small force constant of 0.001 Ha/Bohr^2. The constrained geometry optimization is done with the extremely fast, though inaccurate, GFN-FF[4] level of theory.

Given a set of constraints, molecules may form H-bonds more or less favourably at different relative positions. Thus, we repeated the sampling 2-5 times with multiple initial configurations to generate up to 10000 initial guesses per cluster composition. Inevitably, some of the H-bond combinations would be physically unable to match the constraints without causing unwanted chemical reactions to occur. On average around 50% of combinations are removed as reacted or duplicate structures.

S1.3 XTB Optimization and filtering

After filtering, a relaxed (i.e. without the constraints) geometry optimization is performed at the semi-empirical GFN1-xTB[5] level of theory. This gives a better energy estimate than GFN-FF but is computationally slightly more expensive. Configurations for which hydrogen bonds were either all broken (which we here define as the H$\cdots$O distance rising above 2.2 Å) or converted into covalent bonds (H$\cdots$O distance decreases below 1.4 Å), are filtered out. The ideal O-H$\cdots$O bond angle is around 180 degrees, and the minimum bond angle is set to 120 degrees[3]. We also add N$\cdots$O bonds to the total H-bond count if the N$\cdots$O distance is less than 3.2 Å, and all ON$\cdots$O angles are between 80 and 100 degrees[6].

After the initial low-level sampling, we select the clusters with the highest amount of hydrogen bonds (typically cases where $N_{\mathrm{H-bonds}} \geq N_{\mathrm{donors}}$), and remove duplicate topologies (Section S1.4). Note that intermolecular H-bonds are also added to the total H-bond count, but at least one H-bond must exist between the molecules for the configuration to be considered a cluster. The filtering criteria can be adjusted to select 100-500 cluster configurations for DFT level calculations.

Unfortunately, clusters optimized with cheap semi-empirical methods fail to accurately predict DFT level electronic energies and only allow limited filtering based on energy (see Figure S1). We can obtain a better estimate of the final optimized energy by first running a single-point energy calculation on the XTB optimized structures. This was done using the ωB97X-D3 density functional and def2-SVP basis set, and it allowed us to significantly reduce the energy filtering threshold for further calculations from 15 kcal/mol to 6 kcal/mol (Table 1.). Before geometry optimization at DFT level, we use CREST to improve upon our best configurations.

S1.4 Filtering of duplicate structures

In the standard JKCS uniqueness filtering, clusters are considered duplicates if their energy (E), radius of gyration (g), and dipole moment (μ) are all within some threshold values. (The default thresholds are $\Delta E < 10^{-3}$ Ha, $\Delta g < 10^{-2}$ Å, and $\mu = 0.1$ D.) Particularly for OOM dimers, we found the simple uniqueness criteria to be often insufficient because it fails to provide detailed enough information on the finer cluster structure. Furthermore, they failed to identify several configurations pairs evidently occupying the same potential well, which would likely have been inside the thresholds with different convergence criteria. Thus, we opted for a different strategy more suited for our multifunctional strongly bound OOM clusters: Filtering by H-bond topology.

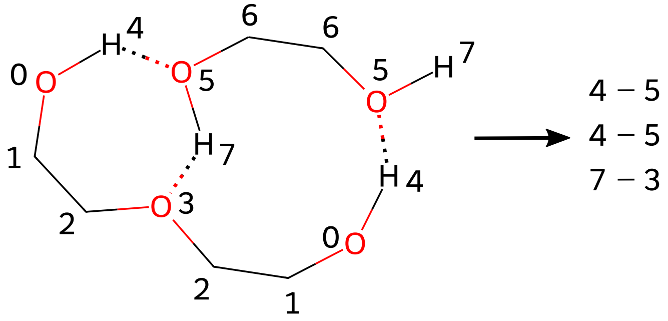

Figure S2: Example graph representation of a $(PEG_1)(PEG_2)$ cluster with atoms labelled by their rank, and the donor-acceptor pairs listed on the right. (Order of the pairs is arbitrary, but sorting is needed to determine identical clusters.)

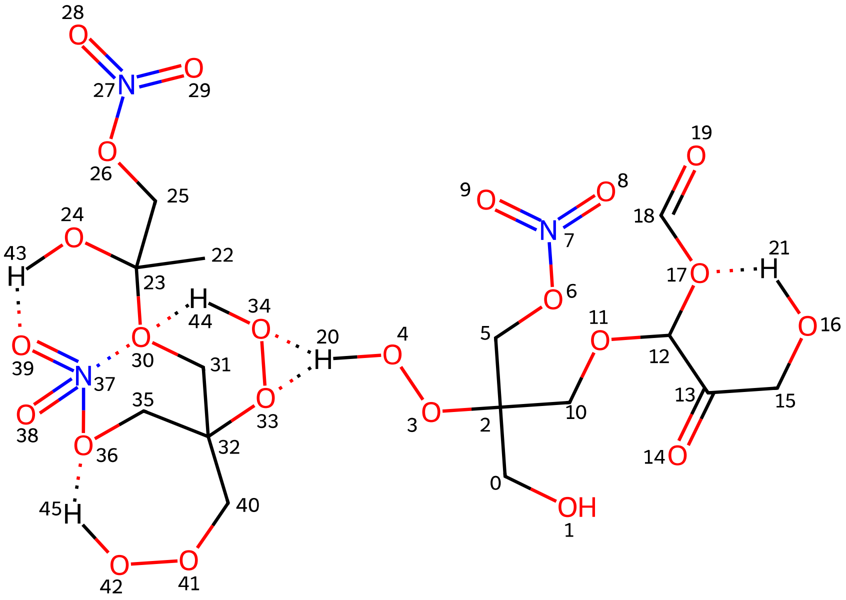

Figure S3: Graph representations of a two-OOM cluster. Only donor hydrogens are shown.

Once all H-bonds and non-covalent N$\cdots$O interactions have been identified using interatomic distance thresholds (1.4 Å < H$\cdots$O < 2.2 Å and 2.8 Å < N$\cdots$O < 3.1 Å, respectively), we can construct a molecular graph description of the clusters and compare whether two cluster graphs are identical (isomorphic). When duplicate topologies are detected, we select only the lowest energy configuration. We illustrate a simple topological descriptor in Figure S2. For a given cluster composition, different configurations can be described compactly as a list of hydrogen bonded donor-acceptor pairs. However, to compare topologies we have to determine the symmetry class of each molecule and label the atoms

by their rank. Most molecular graphs contain symmetries, meaning some atoms or functional groups can be swapped, or the entire molecule can be flipped in a way which results in an identical graph. If the cluster contains two or more identical molecules, we must also test uniqueness when the molecules are swapped. This type of descriptor is sufficient for generating the constraints described in Section S1.2, but it is not unique in all cases.

For duplicate configuration filtering we use the circular/Morgan fingerprint [7], provided in `rdkit` [8], where each bit of the fingerprint corresponds to a fragment within some radius r of a chosen center atom. To ensure uniqueness of the topological fingerprints, the maximum radius is set very high ($r = 20$Å), and the number of each substructure is stored in a count vector. Figure S3 shows a typical cluster graph with atoms labeled by index. The two molecules are held together by H at index 20 bonding with Os at indices 33 and 34 (the bond may or may not be stronger than a singular $O \cdots H$ bond). N at index 37 has also formed a weak bond with O at index 30. However, this cluster is not optimal, due to an unbonded H-donor at index 1. Oxygens in the NO_2-group are treated as interchangeable, i.e. swapping them produces the same fingerprint.

S1.5 Metadynamics (CREST)

While the constrained sampling gives a good estimate for stable configurations, the resulting configurations are likely not at the lowest minima. Slight adjustments to the relative positions of atoms may result in a significantly lower minima. Thus we have used the CREST software [9] to improve upon previously sampled lowest energy cluster configurations at GFN1-xTB level.

The idea of metadynamics [10] is to run a molecular dynamics simulation and add a biasing potential during the simulation preventing the simulation from revisiting previously explored areas of the potential energy surface. CREST is a program developed for low-cost exploration of potential energy surfaces of chemical systems, and is most often used for searching molecular conformers. The conformal search uses an algorithm [11;12], which combines metadynamics simulations and geometry optimizations using the XTB program (i.e. CREST is a program built around XTB).

CREST can be used at multiple points of the workflow. However, we have found it is optimal not to run CREST on every XTB optimized structure, and instead select the lowest structures given by the aforementioned single-point DFT calculation. For All energies are provided in kcal/mol.each low energy structure given, we obtain 10-30 new configurations, although many of these are considered duplicates by our topology criteria (see section S1.4). Another single-point ωB97X-D3/def2-SVP calculation is performed on all the non-duplicate structures generated by CREST, and these are added to the set fed to the next stage of the workflow.

Most OOM-clusters have multiple low energy minima within a few kcal/mol, but some molecules may prefer to cluster in very specific configurations. If the final number of low energy configurations is particularly low (≤ 3) after structure optimization, another CREST run is performed. This is likely to find additional local minima.

S1.6 Final DFT Optimization

After the metadynamics simulations and ωB97X-D3/def2-SVP single point calculations, we used a threshold of $E_{\text{lim}} = 6$ kcal/mol as a selection criteria for DFT optimization (though based on our results even as low as 5 kcal/mol can be sufficient). In some cases, there are still too many structures to optimize after this threshold. In these cases, the best 50 structures were picked (as indicated by N_{lim} in Table 1). In principle, computation time could be further reduced by halting the optimization after 5-10 steps and filtering with a lower threshold value before continuing, but this was not necessary in our calculations.

Electronic energy is a component of the Gibbs free energy, and the variation in G - E between different configurations of the same cluster are limited to around 1-2 kcal/mol (See Figure S1). Thus, computational time can be saved by only calculating frequencies and free energies for a few of the lowest energy clusters (we use $E_{\text{lim}} = 3$ kcal/mol). Low-frequency vibrational modes were corrected using Grimme's quasi-harmonic approximation [13]. The DFT optimization was performed in two stages: First, optimization and frequency analysis was performed using ωB97X-D3/def2-SVP (superscript low), after which a single-point ωB97X-D3/def2-TZVP (superscript high) calculation was performed on all configurations passing a $G_{\text{lim}} = 3$ kcal/mol filter. It was assumed that the lower basis set caused

no significant error to the frequency analysis, in which case the higher-level Gibbs free energy may be approximated with:

$$G^{\text{high}} = G^{\text{low}} - E^{\text{low}} + E^{\text{high}}.$$

Finally, binding free energies were calculated from the lowest energy configuration of dimer and the lowest energy configurations of the isolated monomers:

$$\Delta G = G^{\text{high}}_{\text{cluster}} - \sum G^{\text{high}}_{\text{monomer}},$$

computed at the high DFT level. We found that the single-point corrected values on a low DFT level structure were sufficient to predict the trend observed for PEG dimers (Section 3.2). However, the absolute ΔG values were found to differ from the optimized high level values by up to 2 kcal/mol. Therefore, the lowest OOM-dimers and monoterpene-derived dimers were re-optimized in the def2-TZVP basis, and finally SP corrected with the minimally augmented def2-TZVP basis set [14].

For quantitative results, further energy corrections would be required, including coupled-cluster single-point energy corrections, as well as possible anharmonicity and/or entropy corrections [15;16]. However, especially the coupled-cluster energies are very costly to calculate for large clusters. For the purposes of this study, especially the investigation of relative trends in formation free energies, the high-level DFT results are sufficient.

S2 Dimer binding energies

All energies are provided in kcal/mol.

S2.1 PEGs

Polyethylene glycol Gibbs free binding energies at 298.15 K at the ωB97X-D3/def2-TZVP//ωB97X-D3/def2-SVP level of theory.

Table S1: PEG dimer Gibbs free binding energies.

Cluster	N atoms	Global minimum	Boltzmann average
2EG	20	1.326	1.326
1DEG1EG	27	-1.593	-2.164
2DEG	34	-3.281	-3.165
1TEG1EG	34	-1.564	-1.823
1PEG$_4$1EG	41	-0.872	-0.728
1TEG1DEG	41	-3.740	-3.711
2TEG	48	-0.897	-0.897
1PEG$_5$1EG	48	-2.959	-2.945
1PEG$_4$1DEG	48	1.549	1.684
1PEG$_6$1EG	55	-5.114	-4.981
1PEG$_5$1DEG	55	-4.449	-4.253
1PEG$_4$1TEG	55	2.207	2.439
2PEG$_4$	62	5.962	6.615
1PEG$_7$1EG	62	-4.915	-4.865
1PEG$_6$1DEG	62	-1.141	-1.125
1PEG$_5$1TEG	62	-1.260	-1.147
1PEG$_8$1EG	69	0.846	0.846
1PEG$_7$1DEG	69	-3.742	-3.603
1PEG$_6$1TEG	69	-1.390	-1.251
1PEG$_5$1PEG$_4$	69	0.636	1.134
2PEG$_5$	76	-0.391	-0.233
1PEG$_8$1DEG	76	3.578	3.193
1PEG$_6$1PEG$_4$	76	1.453	1.959
1PEG$_7$1TEG	76	1.524	1.584
1PEG$_6$1PEG$_5$	83	-0.551	-0.671
1PEG$_8$1TEG	83	4.598	4.328
1PEG$_7$1PEG$_4$	83	3.599	3.478
2PEG$_6$	90	-1.963	-1.684
1PEG$_8$1PEG$_4$	90	8.602	8.450
1PEG$_7$1PEG$_5$	90	0.523	0.464
1PEG$_8$1PEG$_5$	97	5.460	5.215
1PEG$_7$1PEG$_6$	97	1.643	1.442
2PEG$_7$	104	3.507	3.372
1PEG$_6$1PEG$_8$	104	6.944	6.700
1PEG$_7$1PEG$_8$	111	3.019	3.033
2PEG$_8$	118	9.939	9.606

S2.2 OOMs

Table S2: Isoprene (Ipr) and Toluene (Tol) accretion products. Nannoolal and SIMPOL saturation vapour pressures at 298 K are in atm.

Name	SMILES	Nannoolal	SIMPOL
IprBBL	CC(OO)(C(ON(=O)(=O)))C(ON(=O)(=O))COC(O)(OO)	7.90E-16	1.47E-13
IprBIH	C(O)C(OO)(C(ON(=O)(=O)))COC(O)(C)C(OO)	2.86E-16	5.57E-14
IprBOM	C(O)C(ON(=O)(=O))C(OO)(C(ON(=O)(=O)))COC(O)C(OO)	3.35E-20	3.63E-16
IprBOW	C(O)C(ON(=O)(=O))C(OO)(C(ON(=O)(=O)))COC(O)C(C)=C	6.40E-16	1.10E-14
IprBRB	CC(O)(C(ON(=O)(=O)))C(OO)COC(O)(C(ON(=O)(=O)))C(OO)	1.51E-19	1.37E-16
IprC	C(O)C(OO)(C(ON(=O)(=O)))COC(C(=O)C(O))OC(=O)	3.40E-16	4.40E-14
IprCMP	CC(O)(C(ON(=O)(=O)))OCC(OO)(C(ON(=O)(=O)))C(OO)	3.08E-16	5.54E-14
IprDEE	CC(OO)(C(ON(=O)(=O)))C(ON(=O)(=O))COC(O)C(OO)	1.54E-16	5.54E-14
IprDK	C(O)C(OO)(C(ON(=O)(=O)))COC(O)(C(ON(=O)(=O)))C(OO)	7.15E-20	3.63E-16
IprDXH	C(O)C(OO)(C(ON(=O)(=O)))COC(O)C(OO)	2.01E-16	1.48E-13
IprEGW	C(O)C(ON(=O)(=O))C(OO)(C(ON(=O)(=O)))COC(O)(C)C(OO)	4.73E-20	1.37E-16
IprFYV	C(O)C=C(C(ON(=O)(=O)))COC(O)C(OO)(C)C(ON(=O)(=O))	2.44E-16	1.10E-14
IprHX	CC(OO)(C(O))COC(O)(C(ON(=O)(=O)))C(OO)	2.86E-16	5.57E-14
IprHZ	CC(OO)(C(ON(=O)(=O)))C(O)COC(O)(C(ON(=O)(=O)))C(OO)	7.12E-20	1.37E-16
IprIGJ	C(=O)(O)C(=O)C(=O)COOCC(=O)C(=O)C(=O)(O)	4.60E-16	1.92E-13
IprNC	CC(OO)(C(ON(=O)(=O)))C(O)OC(C(O))C(C(ON(=O)(=O)))=C	8.33E-16	1.10E-14
IprNN	CC(OO)(C(ON(=O)(=O)))C(O)OC(C(O))C(C)=C(O)	1.00E-15	1.11E-14
IprNV	C(O)C(OO)C(O)(C(ON(=O)(=O)))COC(O)(C)C(ON(=O)(=O))	3.96E-18	2.47E-16
IprQI	CC(OO)(C(O))C(O)OCC(C(ON(=O)(=O)))=CC(O)	2.40E-16	1.11E-14
IprVQ	CC(OO)(C(O))C(O)COC(O)C(ON(=O)(=O))(C)C(ON(=O)(=O))	5.92E-19	9.29E-17
TolA	CC(=C(O))C(O)C=CC(=O)OOC(=O)C=CC(O)C(C)=C(O)	2.40E-16	2.33E-16
TolBGDL	CC(O)(C(=O))C(OO)C(C(O)C(=O))OOc1ccccc1C(=O)	6.28E-17	1.09E-16
TolDOAI	c1cccc(OO)c1COC(O)C(ON(=O)(=O))(C(ON(=O)(=O)))C(=O)	7.96E-17	3.06E-15
TolDYMI	C(=O)c1ccccc1OOC(C)(C(O)C(=O))C(OO)C(O)C(=O)	5.06E-17	1.09E-16
TolDZOQ	C(OO)c1ccccc1OOC(C)(C(=O)C(=O))C(OO)C(O)C(=O)	5.78E-19	1.49E-16
TolEDSJ	c1cccc(O)c1OC(O)(O)C(N(=O)(=O))=CC(=O)(O)	7.82E-17	1.47E-14
TolEDSN	c1cccc(O)c1OC(O)(O)C(N(=O)(=O))=CC(=O)(OO)	6.81E-16	1.78E-13
TolEDSV	C(ON(=O)(=O))c1cccc(N(=O)(=O))c1OC(O)C(O)(OO)	5.29E-17	3.61E-15
TolETDP	C(=O)C(OO)C(O)C(=O)COOCC(=O)C(O)CC(=O)	5.46E-17	7.26E-15
TolETLQ	c1cccc(OO)c1COC(N(=O)(=O))C(=O)C(O)CC(=O)	6.46E-16	2.43E-14
TolETLR	c1ccc(N(=O)(=O))c(O)c1OC(O)C(=O)C(O)CC(=O)	7.50E-16	1.24E-13
TolETLX	c1cccc(OO)c1COC(O)C(=O)C(O)CC(=O)	6.73E-16	2.06E-14
TolEUXV	C(=O)CC(=O)C=CC(=O)OC(=O)C=CC(O)C(O)=C(O)	3.31E-16	3.06E-15
TolEYWZ	c1ccc(N(=O)(=O))c(O)c1OC(O)C(N(=O)(=O))C(=O)(O)	8.06E-17	5.91E-14
TolEYXG	c1cccc(OO)c1COC(O)C(N(=O)(=O))C(=O)(O)	6.17E-17	9.77E-15
TolEYYY	c1ccc(N(=O)(=O))c(OO)c1COC(N(=O)(=O))C(=O)(O)	2.69E-16	3.07E-14
TolFDPZ	C(=O)(O)CC(=O)C(O)COC(=O)C=CC(=O)(O)	7.30E-16	3.86E-14
TolFFVY	c1ccc(N(=O)(=O))c(O)c1OC(O)C=C(N(=O)(=O))C(=O)(O)	1.35E-17	1.73E-14
TolFFVZ	c1cccc(OO)c1OC(O)C=C(N(=O)(=O))C(=O)(O)	4.56E-17	7.62E-15
TolFFWF	C(O)c1ccccc1OC(O)C=C(N(=O)(=O))C(=O)(O)	3.64E-16	5.18E-15
TolFOXV	C(=O)(OO)CC(=O)C(O)COOCC(O)C(=O)CC(=O)(OO)	4.93E-18	1.28E-14
TolFSPZ	c1ccc(N(=O)(=O))c(O)c1OC(O)C(OO)(C(OO))C(=O)	2.04E-19	5.73E-15
TolFZTB	c1ccc(N(=O)(=O))c(O)c1C(=O)OC(O)(N(=O)(=O))C=C(O)	5.72E-16	1.19E-13
TolG	C(O)=CC(O)C(C)=CC(=O)OOC(=O)C=C(C)C(O)C=C(O)	2.40E-16	2.33E-16
TolGECF	c1ccc(N(=O)(=O))c(O)c1C(=O)OOC(=O)c2c(O)c(N(=O)(=O))ccc2	2.70E-16	1.07E-12
TolGECI	C(=O)(O)c1ccccc1OOc2c(N(=O)(=O))cccc2C(OO)	1.87E-16	1.28E-14
TolGECJ	c1ccc(N(=O)(=O))c(OO)c1COOc2ccccc2C(=O)(O)	1.87E-16	1.28E-14
TolKFK	C(OO)C(=O)C=CC(=O)COC(=O)C=CC(=O)C(OO)	1.69E-16	1.21E-12
TolRQG	C(=O)(OO)C=CC(O)C=C(O)COOCC(O)=CC(O)C=CC(=O)(OO)	3.92E-23	3.02E-19
TolZKE	C(OO)C(=O)C(O)C(OO)C(=O)C(OO)	5.67E-18	8.27E-13

All Gibbs free binding were energies calculated using ωB97X-D3 functional at 1 atm and 298.15 K, expect the final column at 223.15 K. Column descriptions for Tables S3-S5:

1. Cluster type
2. ΔG optimized at ωB97X-D3/def2-SVP level of theory
3. Single-point correction with the def2-TZVP basis set
4. ΔG optimized at ωB97X-D3/def2-TZVP level of theory
5. Final SP correction with the minimally augmented def2-TZVP basis set.
6. High level corrected ΔG re-calculated at 223.15 K

Table S3: Isoprene dimer Gibbs free binding energies.

Cluster	low opt	low corrected	high opt	high corrected	low temp
2IprBBL	-5.960	4.255	2.715	3.126	-0.251
2IprBIH	-7.594	-1.536	-1.996	-1.735	-5.415
2IprBOM	-7.870	-1.086	-0.046	0.175	-3.788
2IprBOW	-6.638	0.996	0.010	0.187	-3.565
2IprBRB	-7.170	3.547	1.596	2.195	-1.603
2IprC	-10.665	1.982	-1.747	-0.645	-4.807
2IprCMP	-7.506	2.719	1.224	1.605	-2.629
2IprDEE	-8.530	1.021	-0.391	-0.071	-3.917
2IprDK	-11.707	-0.299	-1.200	-0.614	-4.755
2IprDXH	-7.541	0.174	0.241	0.819	-3.083
2IprEGW	-10.408	0.127	-0.213	0.380	-3.999
2IprFYV	-7.701	5.962	4.329	4.747	0.510
2IprHX	-9.540	-0.005	-0.555	0.147	-3.847
2IprHZ	-8.927	0.589	-1.025	-0.715	-4.546
2IprIGJ	-14.164	-5.313	-6.209	-5.729	-9.364
2IprNC	-12.953	0.572	-0.111	0.687	-4.101
2IprNN	-7.656	0.426	-0.768	-0.283	-3.824
2IprNV	-11.393	1.313	0.935	1.517	-2.503
2IprQI	-8.297	0.732	0.512	1.115	-2.911
2IprVQ	-11.186	-2.196	-3.767	-3.498	-7.251

Table S4: Toluene dimer Gibbs free binding energies.

Cluster	low opt	low corrected	high opt	high corrected	low temp
2TolA	-12.623	-1.853	-3.030	-2.251	-5.733
2TolBGDL	-10.037	0.118	-0.547	0.122	-4.261
2TolDOAI	-12.908	-3.544	-3.772	-3.383	-7.649
2TolDYMI	-8.573	-4.032	-4.405	-4.105	-7.879
2TolDZOQ	-10.031	-1.123	-0.562	-0.242	-4.469
2TolEDSJ	-16.502	-6.653	-7.736	-7.166	-11.257
2TolEDSN	-15.063	-2.426	-3.793	-3.261	-7.697
2TolEDSV	-12.626	-3.261	-4.327	-3.801	-7.556
2TolETDP	-4.957	0.505	0.125	0.603	-3.383
2TolETLQ	-13.633	-4.203	-5.022	-4.597	-8.793
2TolETLR	-7.914	2.548	1.611	2.350	-2.014
2TolETLX	-10.449	-3.042	-3.256	-2.794	-6.852
2TolEUXV	-9.032	1.014	0.192	1.332	-2.600
2TolEYWZ	-13.679	-4.475	-2.916	-2.377	-7.108
2TolEYXG	-10.530	-2.384	-2.646	-2.224	-6.101
2TolEYYY	-14.416	-2.461	-4.613	-4.021	-8.058
2TolFDPZ	-12.197	-5.700	-5.105	-4.827	-8.875
2TolFFVY	-12.642	-3.516	-4.946	-4.404	-8.434
2TolFFVZ	-8.449	-1.894	-3.301	-3.094	-6.755
2TolFFWF	-13.125	-7.362	-6.596	-6.357	-10.138
2TolFOXV	-7.353	-2.049	-2.658	-2.494	-6.111
2TolFSPZ	-4.731	4.313	3.154	3.546	-0.277
2TolFZTB	-6.936	1.275	-0.170	0.236	-3.692
2TolG	-11.282	-1.320	-1.713	-0.891	-5.018
2TolGECF	-7.104	2.831	1.455	1.526	-2.758
2TolGECI	-16.599	-7.066	-6.328	-5.856	-10.476
2TolGECJ	-12.643	-5.610	-6.534	-6.239	-9.940
2TolKFK	-10.351	-1.389	-2.578	-2.208	-5.954
2TolRQG	-13.887	-7.998	-8.197	-7.824	-11.863
2TolZKE	-10.589	-3.303	-3.949	-3.699	-7.284

Table S5: Mixed dimer Gibbs free binding energies.

Cluster	low opt	low corrected	high opt	high corrected	low temp
1IprBIH1IprC	-7.385	3.586	2.057	2.828	-1.164
1IprBIH1IprCMP	-8.101	-2.044	-2.831	-2.606	-6.337
1IprBIH1IprNC	-7.849	1.413	-0.088	0.340	-3.449
1IprBIH1IprQI	-6.525	-0.744	-0.709	-0.479	-4.278
1IprBIH1TolA	-13.109	-3.054	-4.125	-3.287	-7.675
1IprBIH1TolEDSJ	-11.947	-4.111	-5.133	-4.754	-8.661
1IprBIH1TolFFVZ	-10.017	-2.363	-3.324	-2.970	-6.626
1IprBIH1TolFFWF	-7.978	-1.020	-1.412	-1.093	-4.799
1IprBIH1TolG	-8.820	-1.763	-1.677	-1.243	-5.221
1IprC1IprCMP	-9.838	-0.959	-1.727	-1.222	-5.269
1IprC1IprNC	-15.228	-1.856	-3.107	-2.147	-6.639
1IprC1IprQI	-9.513	0.843	0.456	1.314	-2.806
1IprC1TolA	-13.105	0.158	-1.754	-0.586	-4.938
1IprC1TolEDSJ	-13.880	-2.766	-3.827	-3.027	-7.113
1IprC1TolFFVZ	-10.041	0.215	-0.903	-0.200	-4.258
1IprC1TolFFWF	-15.703	-5.868	-6.138	-5.434	-9.491
1IprC1TolG	-9.185	-0.916	-1.918	-1.104	-5.089
1IprCMP1IprNC	-10.085	1.105	-0.055	0.471	-3.794
1IprCMP1IprQI	-9.147	-1.455	-1.092	-0.783	-5.019
1IprCMP1TolA	-10.640	-1.733	-2.484	-1.928	-6.160
1IprCMP1TolEDSJ	-14.477	-3.947	-5.409	-4.912	-9.200
1IprCMP1TolFFVZ	-11.162	-1.819	-2.929	-2.502	-6.563
1IprCMP1TolFFWF	-8.844	-3.404	-3.066	-2.812	-6.622
1IprCMP1TolG	-8.312	2.335	1.365	1.976	-2.208
1IprNC1IprQI	-13.565	-0.548	-0.526	0.111	-4.416
1IprNC1TolA	-14.621	-1.005	-2.190	-1.180	-5.843
1IprNC1TolEDSJ	-16.503	-2.368	-3.361	-2.599	-7.110
1IprNC1TolFFVZ	-13.740	-3.272	-3.319	-2.850	-7.051
1IprNC1TolFFWF	-14.281	-4.589	-4.162	-3.650	-8.031
1IprNC1TolG	-11.479	0.028	-1.311	-0.651	-5.013
1IprQI1TolA	-11.884	-2.353	-2.851	-2.162	-6.640
1IprQI1TolEDSJ	-15.883	-6.287	-6.398	-5.831	-10.122
1IprQI1TolEUXV	-11.697	-1.512	-2.358	-1.543	-5.676
1IprQI1TolFFVZ	-12.800	-3.687	-3.890	-3.428	-7.404
1IprQI1TolFFWF	-14.248	-5.646	-5.519	-5.132	-9.286
1IprQI1TolG	-10.878	-3.615	-3.887	-3.378	-7.422
1IprQI1TolRQG	-6.165	-0.740	-0.559	-0.156	-4.421
1TolA1TolEDSJ	-19.310	-7.381	-8.135	-7.331	-11.835
1TolA1TolEUXV	-15.237	-1.931	-3.596	-2.402	-7.008
1TolA1TolFFVZ	-16.285	-6.285	-7.253	-6.554	-10.772
1TolA1TolFFWF	-15.522	-7.214	-7.234	-6.783	-11.182
1TolA1TolG	-11.999	-0.149	-1.703	-0.759	-5.121
1TolA1TolRQG	-11.349	-4.201	-4.783	-4.303	-8.695
1TolEDSJ1TolEUXV	-13.896	-3.190	-3.956	-2.987	-7.209
1TolEDSJ1TolFFVZ	-12.584	-2.053	-2.787	-2.236	-6.346
1TolEDSJ1TolFFWF	-13.162	-4.384	-4.483	-4.127	-8.204
1TolEDSJ1TolG	-11.915	-2.702	-3.562	-2.903	-7.115
1TolEDSJ1TolRQG	-10.550	-3.026	-6.143	-5.813	-9.655
1TolEUXV1TolG	-11.340	-1.778	-3.075	-2.165	-6.405
1TolEUXV1TolRQG	-7.018	0.281	-0.611	-0.125	-4.127
1TolFFVZ1TolFFWF	-11.614	-2.937	-3.513	-3.245	-7.165
1TolFFVZ1TolG	-17.031	-6.202	-6.926	-6.137	-10.351
1TolFFWF1TolG	-12.113	-4.761	-4.390	-3.904	-8.020
1TolG1TolRQG	-12.685	-3.285	-3.876	-3.331	-7.575

S2.3 Monoterpene-derived accretion product dimers

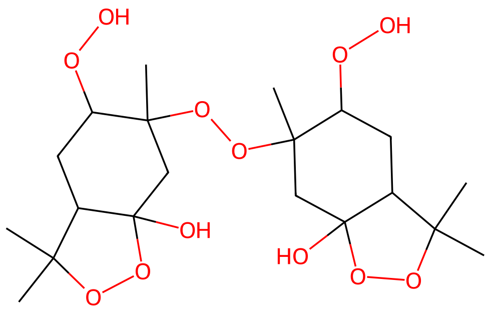

(a) DA

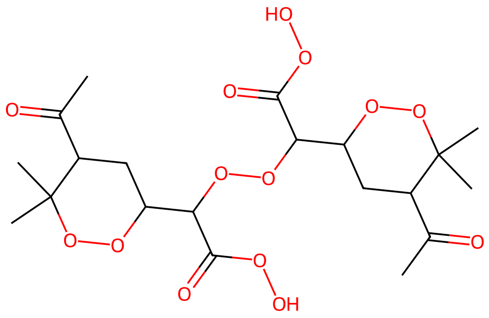

(b) DB

Figure S4: Monoterpene accretion products.

Table S6: Low and high level binding free energies of C20-sized molecule dimers. Temperature is 298.15 K and reference pressure 1 atm.

Cluster	low opt	low corrected	high opt	high corrected	low temp
2DA	-19.277	-4.704	-5.865	-5.084	-9.939
2DB	-16.218	-6.690	-8.856	-8.651	-12.858
1DA1DB	-12.872	-3.173	-5.148	-4.690	-9.093

References

[1] J. Zhang and M. Dolg, *Phys. Chem. Chem. Phys.*, 2015, **17**, 24173–24181.

[2] J. Zhang and M. Dolg, *Phys. Chem. Chem. Phys.*, 2016, **18**, 3003–3010.

[3] E. Arunan, G. R. Desiraju, R. A. Klein, J. Sadlej, S. Scheiner, I. Alkorta, D. C. Clary, R. H. Crabtree, J. J. Dannenberg, P. Hobza, H. G. Kjaergaard, A. C. Legon, B. Mennucci and D. J. Nesbitt, *Pure and Applied Chemistry*, 2011, **83**, 1637–1641.

[4] S. Spicher and S. Grimme, *Angewandte Chemie International Edition*, 2020, **59**, 15665–15673.

[5] C. Bannwarth, E. Caldeweyher, S. Ehlert, A. Hansen, P. Pracht, J. Seibert, S. Spicher and S. Grimme, *WIREs Computational Molecular Science*, 2021, **11**, e1493.

[6] M. Reichel, B. Krumm, Y. V. Vishnevskiy, S. Blomeyer, J. Schwabedissen, H.-G. Stammler, K. Karaghiosoff and N. W. Mitzel, *Angewandte Chemie International Edition*, 2019, **58**, 18557–18561.

[7] D. Rogers and M. Hahn, *Journal of Chemical Information and Modeling*, 2010, **50**, 742–754.

[8] https://github.com/rdkit/rdkit, *RDKit: Open-source cheminformatics*, 2024.

[9] P. Pracht, S. Grimme, C. Bannwarth, F. Bohle, S. Ehlert, G. Feldmann, J. Gorges, M. Müller, T. Neudecker, C. Plett, S. Spicher, P. Steinbach, P. A. Wesołowski and F. Zeller, *The Journal of Chemical Physics*, 2024, **160**, 114110.

[10] A. Laio and M. Parrinello, *Proceedings of the National Academy of Sciences*, 2002, **99**, 12562–12566.

[11] P. Pracht, F. Bohle and S. Grimme, *Phys. Chem. Chem. Phys.*, 2020, **22**, 7169–7192.

[12] S. Grimme, *Journal of Chemical Theory and Computation*, 2019, **15**, 2847–2862.

[13] S. Grimme, *Chemistry – A European Journal*, 2012, **18**, 9955–9964.

[14] J. Zheng, X. Xu and D. G. Truhlar, *Theoretical Chemistry Accounts*, 2011, **128**, 295–305.

[15] J. Kubečka, V. Besel, I. Neefjes, Y. Knattrup, T. Kurtén, H. Vehkamäki and J. Elm, *ACS Omega*, 2023, **8**, 45115–45128.

[16] N. Myllys, J. Elm, R. Halonen, T. Kurtén and H. Vehkamäki, *The Journal of Physical Chemistry A*, 2016, **120**, 621–630.
